# Evidence-Based Tracking of MDR *E. coli* from Bovine Endometritis and Its Elimination by Effective Novel Therapeutics

**DOI:** 10.3390/antibiotics10080997

**Published:** 2021-08-18

**Authors:** Laiba Shafique, Siwen Wu, Amjad Islam Aqib, Muhammad Muddassir Ali, Misbah Ijaz, Muhammad Aamir Naseer, Zaeem Sarwar, Rais Ahmed, Arslan Saleem, Qudratullah, Abdullah Saghir Ahmad, Hongping Pan, Qingyou Liu

**Affiliations:** 1 State Key Laboratory for Conservation and Utilization of Subtropical Agro-Bioresources, Guangxi University, Nanning 530005, China; laibazoologist@gmail.com (L.S.); siwenwu123@163.com (S.W.); panhp65@163.com (H.P.); 2 Department of Medicine, Cholistan University of Veterinary and Animal Sciences, Bahawalpur 63100, Pakistan; 3 Institute of Biochemistry and Biotechnology, University of Veterinary and Animal Sciences, Lahore 54000, Pakistan; muddassir.ali@uvas.edu.pk; 4 Department of Clinical Medicine and Surgery, University of Agriculture, Faisalabad 38000, Pakistan; misbah.ijaz@uaf.edu.pk (M.I.); aamirnaseer167@gmail.com (M.A.N.); 5 Department of Theriogenology, Cholistan University of Veterinary and Animal Sciences, Bahawalpur 63100, Pakistan; zaeemsarwar@cuvas.edu.pk; 6 Central Diagnostic Laboratory, Cholistan University of Veterinary and Animal Sciences, Bahawalpur 63100, Pakistan; raisahmed@cuvas.edu.pk; 7 Department of Geography, Government College University, Lahore 54000, Pakistan; waseer10@gmail.com; 8 Department of Surgery, Cholistan University of Veterinary and Animal Sciences, Bahawalpur 63100, Pakistan; Qudratullah@cuvas.edu.pk; 9 Department of Parasitology, Cholistan University of Veterinary and Animal Sciences, Bahawalpur 63100, Pakistan; abdullahsaghirahmad@cuvas.edu.pk

**Keywords:** cattle, uterine infection, bacteria, antibiotic resistance, synergy testing, field trial

## Abstract

Antibiotic-resistant bacteria have become the predominant etiology of endometritis and thus require effective treatment approaches. We used ultrasonography coupled with clinical signs and presented complaints of reproductive issues to investigate the epidemiology, phylogenetic analysis, antimicrobial resistance, and development of novel therapeutics against *Escherichia coli* isolated from endometritis in bovine (*n* = 304 from 10 commercial dairy farms). The prevalence of bovine endometritis in this study was 43.75%, while among these, 72.18% samples were positive for *E. coli*. Nucleotide analysis performed through BLAST and MEGAX showed 98% similarity to the nucleotide sequence of the reference *E. coli* strain (accession number CP067311.1). The disk diffusion assay revealed pathogen resistance to most antibiotics. Pattern of MIC order of resistance was as follows: enrofloxacin < gentamicin < co-amoxiclav < streptomycin < amoxicillin < metronidazole < oxytetracycline. Field trials revealed the highest recovery rate (in terms of clearance of endometritis and establishment of pregnancy) in case of gentamicin + enrofloxacin (100%) and gentamicin alone (100%), followed by co-amoxiclav + gentamicin (84.61%), oxytetracycline alone (78.57%), and metronidazole + enrofloxacin (33.33%). Hence, the current study reported a higher prevalence of multidrug-resistant *E. coli* showing considerable similarity with reference strain, and finally, the effective response of novel antibiotics to treat cases.

## 1. Introduction

Reproduction is the fundamental characteristic of all animals, which is essential for the survival of their species [1]. Reproductive diseases such as pyometra, metritis, endometritis, retained fetal membrane (RFM), and general uterine infections are dominant deteriorating challenges that affect the fertility of dairy animals [2]. Metritis and endometritis play a vital role in causing infertility, lower performance, early culling, and genetic loss in dairy cows [3]. Clinical endometritis is defined as the presence of purulent or mucopurulent vaginal discharge postpartum (3 weeks or later) or postnatal discharge. Subclinical endometritis is defined as the infection of the endometrium diagnosed by the presence of neutrophils in the biopsy and histological investigations in the absence of clinical signs of endometritis [4,5].

Bacterial infections and the mechanism of the local immune system of the uterus are important determinants in the prognosis of endometritis. *Escherichia coli (E. coli)* and *Trueperella pyogenes* have been reported to be the most common pathogens isolated from the bovine uterus [6,7,8]. *E. coli* (Gram-negative bacteria) is equally important for livestock and human health and results in a wider range of infections, while its resistance against antibiotics is still debatable [9,10]. The rapid development of resistance, harmful effects of antibiotics, and less availability of antibiotic options are salient issues [11] that are the outcome of four mechanisms of bacteria, i.e., inactivation of bacterial site, active efflux of bacteria, modification of target sites, and altered metabolic pathways by the bacteria [12].

The existence of broad-spectrum β-lactamase-producing *E. coli* in dairy animals has been reported in various studies [13]. Before the advent of the antibiotic era, postpartum ailment was an important reason for maternal death, while a sharp decrease in maternal morbidity was observed upon the use of antibiotics. An appropriate treatment will not only decrease maternal morbidity but may also improve antibiotic stewardship [14]. However, there are limited options of antibiotics to be used in combination [15] to combat the bacterial challenge. The rise in antimicrobial resistance owes to the continuous use of antibiotics that finally become ineffective due to genetic variations in the bacteria [16]. Thus, the study hypothesis states, “*E. coli*-based endometritis is prevalent along with multiple drug resistance, and newer antibiotic approaches are effective against to treat *E. coli*-based endometritis”. The hypothesis was tested with the objectives of investigation of epidemiology, phylogenetic analysis, antimicrobial resistance, and development of new therapeutic approaches against *E. coli* isolated from endometritis in bovine.

## 2. Materials and Methods

### 2.1. Tracking Bovine Endometritis

**Figure 1:**
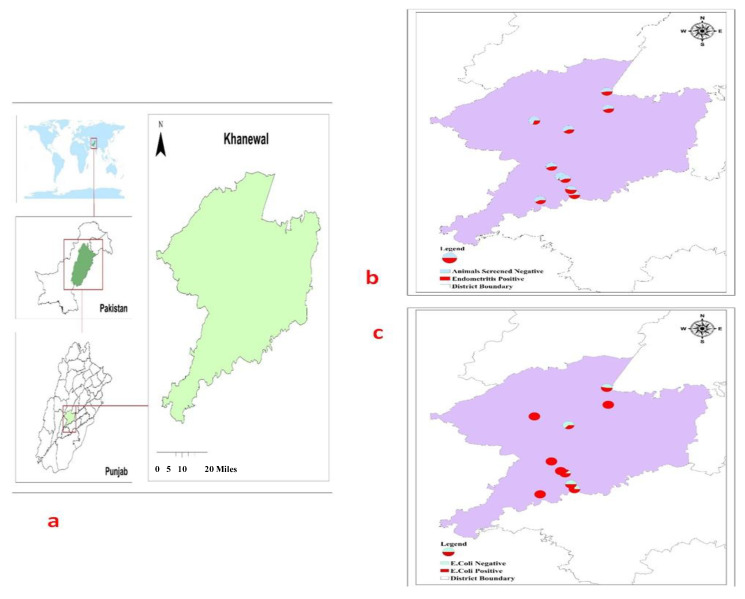
Tracking maps of study area, endometritis, and *E. coli.* (**a**) Green map indicates area of study from where samples were collected. (**b**) Number of positive endometritis cases indicated in red in the circles. Each circle is a complete farm. (**c**) Prevalence of *E. coli* indicated in red (circle). Each circle indicates individual dairy farm. Red area of the circle is indicative of positive samples.

**Figure 2:**
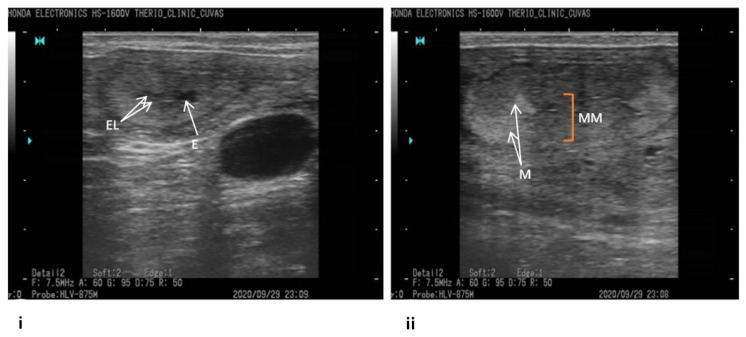
Ultrasonographic pictures showing endometritis in cattle. (**i**) EL—endometrial lining; E—endometrium, white arrows indicate fluid; (**ii**) MM—myometrium; M—metrium, white arrows indicate pus.

The accessible commercial dairy farms big enough to accommodate at least 50 active lactating dairy cows, endometritis identified through ultrasonography, and availability of professional veterinary supervision were the inclusion criteria for this study [17,18]. Keeping in view the set guidelines, commercial dairy farms (*n* = 10) from Khanewal district, Punjab, Pakistan, were selected for this study (Figure 1). Using a convenient sampling technique, *n* = 304 exotic cows (Holstein Frisien) from these farms were included in this study [19]. An ultrasound machine (B mode, 7.5 MHz linear array trans-rectal probe) was used to visualize the reproductive tract (Figure 2), and endometritis was characterized by distended lumen filled with partially echogenic snowy patches as recommended by Fissore et al. The fluid in the uterine environment is indicated black (non-echogenic) while the inflamed uterus, having pus cells, appears with distended lumen filled with whitish-grey areas (echogenic) as referred by Pearson et al. and Fissore et al. [20,21]. When some rays are passing through the structure, and many are coming back that appear hypoechoic (grey scale). If all rays are reflecting back from any structure, it appears hyperechoic (whitish), as referred by B Ihnatsenka et al. [22]. In the case of endometritis, pus is present inside the uterine lumen, due to which some ultrasound rays pass through it and some reflect back, and it appears as a mixture of hypo and hyperechoic images. Aseptically uterine flushing was done using an artificial insemination gun, and fluid was aspirated through a syringe and collected in a sterile sheath. The collected samples were shipped to Central Diagnostic Lab (CDL) of the Cholistan University of Veterinary Sciences, Bahawalpur, Pakistan, maintaining a cold chain at 4 °C.

### 2.2. Isolation and Confirmation of E. coli

The uterine samples were first incubated in nutrient broth for 24 h at 37 °C, while later, these samples were centrifuged at 6000× *g* for 10 min. The sediments were swabbed on blood agar and incubated at 37 °C for 24 h [23]. Harvested microbial colonies were streaked on MacConkey agar, and the resulting growth was subjected to a series of biochemical tests (gram staining, coagulase, and IMViC test), according to the guidelines of the Bergey’s Manual of Determinative Bacteriology [24].

### 2.3. Molecular Confirmation of E. coli

**Figure 3:**
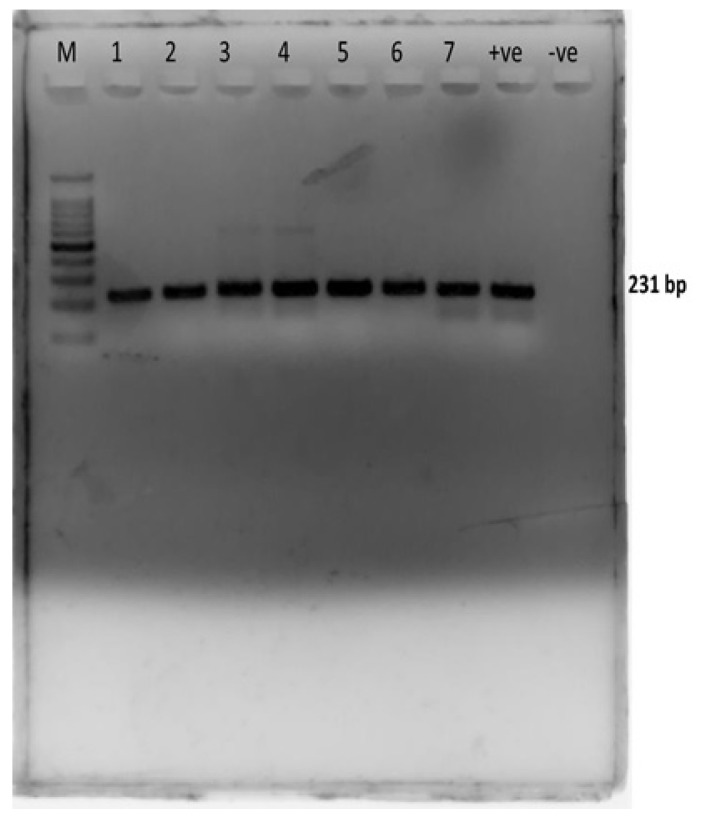
Genotypic confirmation of *E. coli* from endometritis samples. M = marker 1000 bp, wells 1–7 Sample at 231 bp, +ve = positive control, −ve = negative control.

For molecular confirmation of *E. coli*, PCR amplification was done using the *E. coli* 23S rRNA gene-specific primers (E23S-F: ATCAACCGAGATTCCCCCAGT; E23S-R: TCACTATCGGTCAGTCAGGAG), with amplification of 231 bp product (Figure 3). PCR conditions were as follows: denaturation at 95 °C for 3 min, followed by 35 cycles of 95 °C for 1 min, 60 °C for 1 min, and 72 °C for 1 min, and a final extension step at 72 °C for 7 min. The obtained PCR products were subjected to 2% agarose gel electrophoresis [25].

#### 2.3.1. Sequencing of the Local *E. coli* Isolate

The sequence was submitted to NCBI (MZ344556). The sequencing results of the local *E. coli* isolate were analyzed using different bioinformatics tools. Nucleotide analysis of the nucleotide sequences was performed using BLAST to check the similarity with reference NCBI strain. The phylogenetic tree of nucleotide sequences was constructed using the MEGAX software by the neighbor-joining method [26]. Different (21) sequences were also involved in phylogenetic analysis of the nucleotide sequences of *E. coli* 23S ribosomal RNA gene. The tree was drawn to scale, with branch lengths in the same units as those of the evolutionary distances used to infer the phylogenetic tree. The evolutionary distances were computed using the maximum composite likelihood method and were in the units of the number of base substitutions per site.

#### 2.3.2. Gene Structure and Motifs Elicitation

Additionally, conserved motifs of *E. coli* were explored using the MEME Suite (Multiple EM for Motif Elicitation) [27]. The 23S *E. coli* ribosomal RNA gene sequence isolated from bovine uterine samples from Pakistan was submitted in the MEME Suit for motif evaluation. Gene structure display server 2.0 was used for the structural analysis of the desired gene to visualize the exonic regions in the submitted data.

### 2.4. Assessment of the Antibiotic Resistance Profile of E. coli

Disc diffusion assay was used to access the antibiogram of previously confirmed isolates (*n* = 50) against commonly used antibiotics as per the Clinical and Laboratory Standards Institute (CLSI) guidelines [28]. The representative sample size of isolates was enrolled in such a way that *n* = 5 *E. coli* samples from each dairy farm (endometritis positive cows) were randomly selected, making a total of *n* = 50 for in vitro trials. The antibiotics tested included fusidic acid (10 µg), enrofloxacin (10 µg), ciprofloxacin (5 µg), trimethoprim-sulfamethoxazole (25 µg), Amoxicillin, chloramphenicol (30 µg), vancomycin (30 µg), gentamycin (10 µg), linezolid (30 µg), and cefoxitin (30 µg). Briefly, antibiotic discs were aseptically placed on pre-seeded cation-adjusted Mueller-Hinton agar plates. Following incubation for 24 h, the zone of inhibition around each antibiotic disc was compared with the standards provided by CLSI [28].

### 2.5. In Vitro Therapeutic Testing of Antibiotics

Commonly used antibiotics (alone/in combination) were tested against resistant *E. coli* isolates. In vitro testing was performed, initially using the well diffusion test and later using the broth microdilution method to determine synergistic combinations among a range of drugs, including co-amoxiclav, enrofloxacin, metronidazole, oxytetracycline, gentamicin, streptomycin, and amoxicillin. Well diffusion assay identified comparative activity of different drugs while minimum inhibitory concentration (MIC) was performed to authenticate their activity. MIC of individual drugs and drugs in combination was dealt with in the synergy testing section to find the best-suited combination for the field trial.

#### 2.5.1. Well Diffusion Assay

Wells of 6–8 mm in diameter were dug on pre-seeded Mueller-Hinton agar plates using a well borer. Antibiotics to be tested (alone or in combination) were poured into the wells to assess their antibacterial potential. Overnight incubation at 37 °C was followed by measuring of zones of inhibition to determine synergistic drug combinations [29].

#### 2.5.2. Synergy Testing Using Broth Dilution Method

Drugs combinations against resistant *E. coli* isolates (1–1.5 × 10^5^ CFU/mL) were assessed for their synergy using the checkerboard method. The optical density of 96 well plates was measured at 570 nm before and after 24 h incubation at 37 °C. Inhibitory concentration indices were measured as per the formula given below. To avoid errors, the experiment was performed in triplicate [29,30].

FICI = FIC of product A + FIC of product B

FIC of product A = MIC of product A in combination with product B/MIC of product A alone

FIC of product B = MIC of product B in combination with product A/MIC of product B alone

An FICI of ≤0.5 was considered as synergistic, >0.5 but ≤1.0 as an additive, >1.0 but <4.0 as indifferent, and >4.0 as antagonistic.

### 2.6. Field Evaluation of In Vitro Outcomes

Animals positive for *E. coli*-based endometritis, irrespective of the involvement of other bacteria, were selected for the evaluation of in vitro results of the study. The drugs that showed promising results in the in vitro investigations were tested in the field trial. The drug combinations and dosage regimens are shown in Supplementary Materials Table S1. The success rate was determined on the basis of the reproductive indices and physical observations such as disappearance of ailment signs in ultrasonography, negative culture results of uterine flushing, and establishment of pregnancy [18,31].

### 2.7. Statistical Analysis

Descriptive statistics such as univariate analysis were used for the analysis of prevalence and antibiotic susceptibility [32]. On the other hand, parametric tests such as *t*-test (to compare means of two groups) and ANOVA (to compare means of more than two groups) were applied to data obtained in quantitative form. Tukey test as a post hoc test following ANOVA was applied to find significant differences among different groups. Percentage increase in zone sizes and fractional inhibitory indices were calculated using the prescribed formulas [29,30]. The statistical analysis was carried out using the computer-based statistical program SPSS (version 20) at a 5% probability level.

## 3. Results

### 3.1. Prevalence of Endometritis and E. coli

The current study showed an overall prevalence of endometritis at 43.75%, while *E. coli* were 72.18% among these from different farms (Table 1).

### 3.2. Sequencing Results of the Local E. coli Isolate

#### 3.2.1. Nucleotide Analysis

Nucleotide analysis revealed that the 23S rRNA nucleotides sequence of the local *E. coli* isolate showed 98% similarity to the nucleotide sequence of the reference *E. coli* strain (accession number CP067311.1) (Supplmentary Materials Figure S1).

#### 3.2.2. Phylogenetic Analysis

The evolutionary history was inferred by using a phylogenetic tool. Two clades were observed in the phylogenetic tree (Figure S2), which was obtained after comparing the 23S ribosomal RNA gene (nucleotide sequences) of the local *E. coli* isolate with the other sequences available in the NCBI database. The nucleotide sequence of the 23S rRNA gene of the local *E. coli* isolate was found to be closely related to that of the *E. coli* isolate from human rectal swab (Laos) and was clustered together with the *E. coli* isolated from human rectal swab (Laos), human urine (USA), swine liver (China), dairy manure (Canada), human feces (Singapore), chicken meat inner strip (Romania), human urine (China), human (Spain), and pig feces (Canada). The percentage of replicate trees wherein the associated taxa clustered together in the bootstrap test (1000 replicates) was indicated next to the branches. In total, there were 211 positions in the final dataset after the removal of ambiguous positions from each sequence pair.

#### 3.2.3. Gene Structure and Motif Analysis

To gain further insights into the 23S gene of *E. coli*, gene structure and motif analysis were predicted. Five motifs were identified on different positions in 23S rRNA gene sequence. E. value, sites and width of motifs is given in Figure S3. *p*-value (2.49 × 10^−100^) is the same for all five sequences (Figure S3). The position of motif 1 is from nucleotide 1 to 29, as shown in Figure S3 (i). The position of motif 2 is from nucleotide 40 to 89 Figure S3 (ii). The position of motif 3 is from nucleotide 106 to 155, as demonstrated in Figure S3 (iii). The position of motif 4 is from nucleotide 163 to 212, as shown in Figure S3 (iv). The position of motif 5 is from nucleotide 213 to 233, as shown in Figure S3 (v). Frequencies of nucleotides of 23S RNA gene are given in Table 2. Figure S4 shows the structural analysis of the 23S *E. coli* ribosomal gene coding region of Pakistan revealed that this gene possesses the same pattern of the coding region of *E. coli* 23S ribosomal gene isolated from different samples of *E. coli* from different countries.

### 3.3. Antibiogram of Endometritis-Originated E. coli

**Figure 4:**
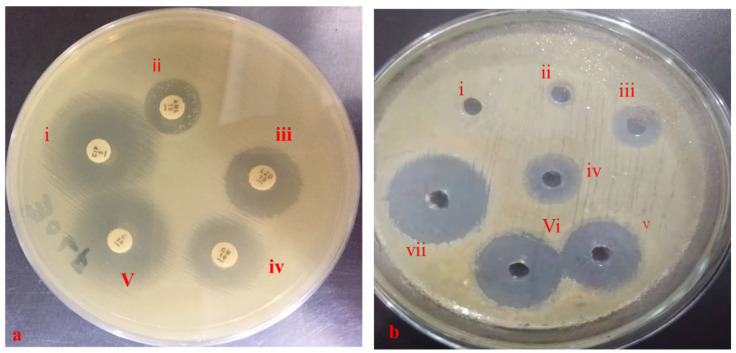
Antibacterial activity of different antibiotics/drugs against *E. coli* using disc diffusion and well diffusion test. (**a**) Antibiotic disc diffusion. i—Ciprofloxacin; ii—Amoxicillin; iii—Linezolid; iv—Gentamicin; v—Chloramphenicol. (**b**) Well diffusion test. i—no zones, negative control; iv—Amoxicillin alone; iii—Enrofloxacin alone; ii—metronidazole; v—Gentamicin + Amoxicillin; vi—Amoxicillin + Enrofloxacin; vii—positive control.

The resistive response of *E. coli* isolates against different antibiotics was in the following order: fusidic acid > vancomycin > amoxicillin > linezolid > cefoxitin > gentamicin ≥ enrofloxacin > ciprofloxacin ≥ trimethoprim-sulfamethoxazole ≥ chloramphenicol (Table 3). The resistance of *E. coli* against fusidic acid, vancomycin, and amoxicillin was 80%, 70%, and 50%, respectively, whereas none of the isolates was resistant against ciprofloxacin, trimethoprim-sulfamethoxazole, and chloramphenicol. The antibacterial activity of different antibiotics is presented in Figure 4a.

### 3.4. In Vitro Therapeutics of Commonly Used Anti-Microbials

#### 3.4.1. Wells Zones of Microbial Growth Inhibition

The drugs, alone and in combinations, showed varying responses against *E. coli*. Inhibition zones of co-amoxiclav, enrofloxacin, metronidazole, amoxicillin, and streptomycin did not differ significantly when compared to their combinations (*p* > 0.05), while gentamicin and oxytetracycline showed significant differences (*p* < 0.05) when compared to their respective combinations (Table 4, Figure 4b).

**Figure 5:**
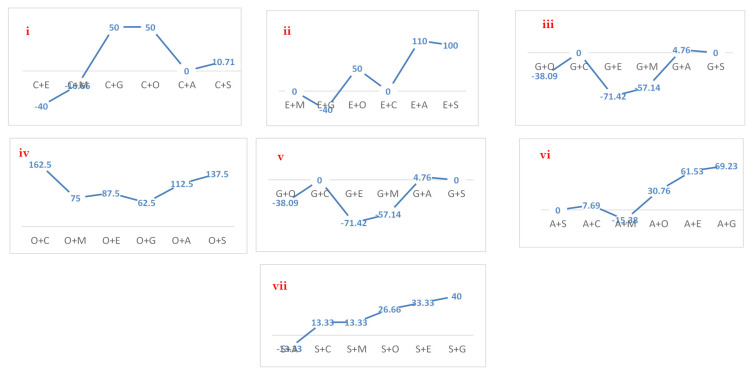
Percentage variation (increase/decrease) in zone of inhibition (ZOI) of individual drugs when compared with their combinations with other drugs. (**i**)—% variation in ZOI of Co-amoxiclav (C) when it was compared with its combination with other drugs; (**ii**)—% variation in ZOI of Enrofloxacin (E) when it was compared with its combination with other drugs; (**iii**)—% variation in ZOI of Gentamicin (G) when it was compared with its combination with other drugs; (**iv**)—% variation in ZOI of Oxytertracylin (O) when it was compared with its combination with other drugs; (**v**)—% variation in ZOI of Metronidazole (M) when it was compared with its combination with other drugs; (**vi**)—% variation in ZOI of Amoxicillin (A) when it was compared with its combination with other drugs, (**vii**)—% variation in ZOI of Streptomycin (S) when it was compared with its combination with other drugs.

Drug combination analysis via well diffusion assay revealed a maximum increase in the zone of inhibition (162.5%) for the oxytetracycline + co-amoxiclav combination in comparison to that of oxytetracycline (Figure 5). Similarly, increase in the zone of inhibition for metronidazole + oxytetracycline, metronidazole + co-amoxiclav, oxytetracycline + enrofloxacin, oxytetracycline + metronidazole, and oxytetracycline + gentamicin was 133.33%, 100%, 87.5%, 75%, and 62.5%, respectively, in comparison to that of the first antibiotic in each combination. Drug activities against *E. coli* using the well diffusion method are shown in Figure 4b.

#### 3.4.2. Synergy Testing of Anti-Microbials

The MIC of the tested drugs varied significantly in the following order: enrofloxacin < gentamicin < co-amoxiclav < streptomycin < amoxicillin < metronidazole < oxytetracycline. All isolates were susceptible to enrofloxacin (2.20 + 1.37) and gentamicin (3.02 + 2.55) (Supplementary Materials Table S2).

Synergy testing of drug combinations against *E. coli* isolates showed no synergistic effect, while antagonism was noted for the combination of enrofloxacin with oxytetracycline. An additive effect was observed among the following combinations: amoxicillin + metronidazole, amoxicillin + gentamicin, amoxicillin + streptomycin, co-amoxiclav + gentamicin, co-amoxiclav + enrofloxacin, and co-amoxiclav + metronidazole. However, no additive effect was noted in the following combinations co-amoxiclav + metronidazole, co-amoxiclav + streptomycin, co-amoxiclav + enrofloxacin, metronidazole + gentamicin, and metronidazole + oxytetracycline (Table 5).

### 3.5. Field Trial Outcome

**Figure 6:**
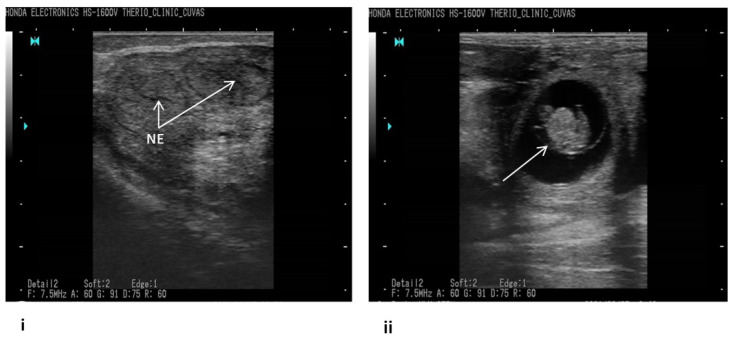
Ultrasonographic pictures after treatment. (**i**) NE = normal endometrium. (**ii**) Arrow indicates the fetus as a sign of established pregnancy.

The highest recovery rates were noted for gentamicin + enrofloxacin (100%), gentamicin alone (100%), co-amoxiclav + gentamicin (84.61%), oxytetracycline alone (78.57%), and metronidazole + enrofloxacin (33.33%), with the success rate represented in terms of normalization of the uterine wall and pregnancy establishment (Figure 6). On the other hand, amoxicillin+ streptomycin, amoxicillin + metronidazole, and amoxicillin + gentamicin showed 15.33%, 10.50%, and 9.50%, respectively, success rate in the field trial indicating a difference of response to that of exhibited in vitro response among *E. coli*.

## 4. Discussion

*Escherichia coli* is an opportunistic bacterial pathogen, well known for its intrinsic and acquired resistance and ability to cause serious infections in animals [33]. Recently, 21 resistance pathways in *E. coli* have been identified against antibiotics [34], but a particular focus on *E. coli* from uterine samples is found in fewer than needed studies [35], despite its frequent isolation from endometritis [36]. Although wider ranges of Gram-positive and Gram-negative bacterial species are expected from endometritis [37], *E. coli* has been found as a common etiology [38]. The higher prevalence of endometritis and isolation of *E. coli* as the most prominent pathogen in the current study is in line with findings from a previous study [38]. The study found 83.33% endometritis, and from these samples, *E. coli* stood at 36.66%, appearing as the most prevalent pathogen. In contradiction to the current study, 26% of Japanese Holstein cattle were positive for endometritis, whereas the prevalence of *E. coli* stood as the most prominent microbe [2]. In another study, in agreement with the current study, the bacteriological investigations of uterine flushing of Holstein cattle showed *E. coli* as the most common isolate [39].

Pattern motifs in the current study were found similar to those reported on different species in literature by using MEME [27,40,41]. The present study revealed that the 23S rRNA nucleotides sequence of the local *E. coli* isolate showed 98% similarity to the nucleotide sequence of the reference *E. coli* strain (accession number CP067311.1). Gene sequencing has been proven to be a reliable conformational genetic marker as it is present in all bacteria, and its function has not changed over time [41,42,43]. Sanger sequencing of the variable gene (23S rRNA) was used for bacterial identification [42,44]. As defined in the CLSI guidelines, species identification can be assigned when the maximum score is 99% or higher and if the sequence similarity between the best and second-best species is greater than 0.5% using DNA target sequencing. Similar type of studies on different pathogens (*Staphylococcus aureus*, *Pseudomonas aeruginosa*, and *E. coli*) have been reported by [45,46,47] as a suitable tool for identification.

The current study was in line with findings of other studies in estimating the resistance of this *E. coli* to ciprofloxacin, i.e., 80%. The high resistance of *E. coli* against these antibiotics indicates the misuse and overuse of these drugs in hospitals and clinics without any cultural investigations [48]. Antibiogram of *E. coli* isolates from cattle depicted high resistance to amoxicillin and penicillin G, whereas good susceptibility was seen for amoxicillin and ciprofloxacin. Emerging resistance of the pathogen to chloramphenicol, gentamicin, and oxytetracycline is alarmingly evident [49]. Gentamicin resistance in *E. coli* has also been reported by Sabat et al. [40]. Additionally, high chloramphenicol resistance in the *E. coli* isolates that originated from livestock was reported previously [50]. It has also been reported that *E. coli* isolated from buffaloes showed high resistance to amoxicillin [51]. Resistance to these antibiotics is thought to be because of the O158:NM strain of *E. coli* [52]. The *E. coli* isolates of dairy origin showing high resistance to penicillin, cephalosporin, and enrofloxacin are in line with previous studies [41,43,44,53]. Resistance in *E. coli* is attributed to *eae-A, F-41, stx-I*, and *stx-II* genes, as reported in some studies [54,55,56,57]. In addition to this, production of extended-spectrum beta-lactamase and *bla* gene groups, most common of which are *bla_CTX-M15_, bla_CTX-M55_, bla_CTX-M14_*, *ST-410*, *ST-23* complex, *ST-10*, and *ST-167* genes [58]. MICs of enrofloxacin and gentamicin of the current study is in line with [59] and is supposed to be because of plasmid-based evolutionary pathways of microbial resistance [60]. The MIC of gentamicin was reported as 1 µg/mL (MIC 50), while that of chloramphenicol was 8 µg/mL against Gram-negative bacteria [61].

Antimicrobial resistance modulation by combination of antibiotics with non-steroidal anti-inflammatory drugs [29,30,62], plant extracts [63,64], and nano-particles [65,66] is well-documented with promising results. The combination of penicillin and gentamicin resulted in a 61% reduction in the MIC value of penicillin with a 100% cure rate against drug-resistant pathogens [67]. The current study showed an enhancement of zones of inhibition when certain antibiotics were used in combination were in agreement with recent studies [68,69]. Carfora et al. [70] reported very lower percentages (1.3%) of resistant isolates to combination therapy. Similar variability trends in the outcomes of in vitro and in vivo investigations, noted in the current study, were also reported in the literature [68,69,71,72]. Temperature variations, cellular binding pathways, the influence of cell matrix on drug action [71], local immunogenic response, optimization and validation of in vivo model to minimize individual variations, enzymatic degradation, activity stabilization [68], pharmacokinetic and pharmacodynamics parameters of the living body [72] are the contributing factors which cause alterations in laboratory and in vivo results; thus, making it difficult to achieve the reproducibility and repeatability of outcomes [73]. The synergy of antibiotics depends on active sites for drugs and how the bacteria respond to different antibiotics. Penicillin groups act on protein binding site 3, while cephalosporins in protein binding site 1. Amino glycosides bind to 30S ribosome subunits, inhibiting the migration of peptide-tRNA from site A to P, resulting in the misreading of mRNA. Antagonism between bacteriostatic and bactericidal antibiotics is also an obvious factor [74] that might be the reason for the failure of some of the drug combinations to inhibit *E. coli*. AS, antimicrobial resistance is the evolutionary mechanism under the effect of a constantly changing environment [12]. The poor response of enrofloxacin in this study might be because of the repeated use of this antibiotic in the study area. It was found on investigation that enrofloxacin had long been used in various health issues in the study region. Gentamicin is suggested to be used as an effective drug without enrofloxacin to avoid further resistance. Moreover, the current study focused on *E. coli*-based endometritis while there are significant chances of multiple bacterial etiologies that complicate cases. In that context, the outcome of this study may or may not be effective because the responses from multiple bacteria might be different. Studies are needed to address epidemiological and genetic discrepancies associated with the spread of such resistant microbes in routine dairy analysis. Similarly, antibiotics must be evaluated against other pathogenic bacterial isolates of dairy origin [18].

## 5. Conclusions

The study results showed a high prevalence of endometritis with context to *E. coli* in dairy cattle. The pathogen not only proved to be multidrug-resistant in an antibiogram trial but also showed substitution and addition of amino acids upon nucleotide sequence analysis. Additive drug interaction was noted when amoxicillin was combined with metronidazole, streptomycin, and gentamicin. The same response was observed in case of co-amoxiclav + gentamicin, enrofloxacin + gentamicin, and enrofloxacin + metronidazole. There was only one antagonism found when enrofloxacin was used in combination with oxytetracycline. The highest success rate in the field trial was observed in the case of application of gentamicin alone and in combination with enrofloxacin, followed by were co-amoxiclav + gentamicin, oxytetracycline alone, and metronidazole + enrofloxacin. This study thus standardizes the endometritis treatment regime by addressing in vitro as well as in vivo parameters of commonly used antibiotics and, thus, paves the way for further investigation on the behavior of *E. coli* at the molecular level. The study also recommends rotational use of antibiotics and additional focus on multiple bacterial etiologies to find the solution for complicated cases.

## Figures and Tables

**Table 1:** Prevalence of endometritis and *E. coli*.

Farm Name	Prevalence of Endometritis				Prevalence of *E. coli*		
	No. of Animals Screened (A)	Endometritis Positive (B)	Prevalence (B/A × 100)	Confidence Interval (95%)	*E. coli* Positive (C)	*E. coli* % (C/B × 100)	Confidence Interval (95%)
Usama Dairies	27	9	33.33	18.64–52.17	9	100	70.09–100
Sahu Dairies	27	8	29.63	15.85–48.48	8	100	67.56–100
United Dairies	18	8	44.44	24.56–66.28	8	100	67.56–100
Rajpoot Dairies	34	14	41.18	26.37–57.78	5	35.71	16.34–61.23
Horizon Dairies	54	26	48.15	35.4–61.15	19	73.08	53.92–86.3
Abdullah Dairies	12	5	41.67	19.33–68.05	5	100	56.55–100
Masab Dairies	28	13	46.43	29.53–64.19	7	53.85	29.15–76.8
Sifari Dairies	48	26	54.167	40.29–67.43	13	50	32.06–67.94
Sial Dairies	38	16	42.10	27.86–57.81	14	87.50	63.98–96.5
Hiraj Dairies	18	8	44.44	24.56–66.28	8	100	67.56–100
Total	304	133	43.75		96	72.18	

**Table 2:** Frequencies of purines and pyrimidines in 23S gene sequence.

Nucleotide	Frequency
A	0.227
C	0.273
T	0.227
G	0.273

**Table 3:** Antibiogram of *E. coli* isolates.

Antibiotics	Potency (µg)	Resistant (%)	Intermediate (%)	Sensitive (%)
Fusidic acid	10	80	20	0
Enrofloxacin	10	10	20	70
Ciprofloxacin	5	0	30	70
Trimethoprim Sulfamethoxazole	25	0	10	90
Amoxicillin	5	50	20	30
Chloramphenicol	30	0	30	70
Vancomycin	30	70	30	0
Gentamicin	10	10	40	50
Linezolid	30	40	30	30
Cefoxitin	30	30	30	40

**Table 4:** Comparison of zones of inhibitions (mm) of *E. coli* isolates against different combinations of antibiotics/drugs.

Drugs/Antibiotics Used		Mean ± Std.	*p*-Value
Drug’s Name	Combination of Drugs		
Co-amoxiclav	Alone	7 ± 1.414	0.073
	C + E	5 ± 1.414	
	C + M	6 ± 2.828	
	C + G	10.5 ± 2.121	
	C + O	10.5 ± 0.707	
	C + A	7.0 ± 1.414	
	C + S	8.5 ± 0.707	
Enrofloxacin	Alone	5 ± 0.00	0.162
	E + M	5 ± 1.414	
	E + G	3 ± 0.00	
	E + O	7.5 ± 2.121	
	E + C	5 ± 1.414	
	E + A	10.5 ± 6.364	
	E + S	10.0 ± 1.414	
Metronidazole	Alone	3 ± 0	0.246
	M + G	4.5 ± 0.707	
	M + O	7 ± 1.414	
	M + C	6 ± 2.828	
	M + E	5 ± 1.414	
	M + A	5.5 ± 0.707	
	M + S	8.5 ± 3.535	
Oxytetracycline	Alone	4 ± 1.414	0.016
	O + C	10.5 ± 0.707	
	O + M	7 ± 1.414	
	O + E	7.5 ± 2.121	
	O + G	6.5 ± 0.707	
	O + A	8.5 ± 0.707	
	O + S	9.5 ± 0.707	
Gentamicin	Alone	10.5 ± 0.707	0.001
	G + O	6.5 ± 0.707	
	G + C	10.5 ± 2.121	
	G + E	3 ± 0	
	G + M	4.5 ± 0.707	
	G + A	11.0 ± 1.414	
	G + S	10.5 ± 0.707	
Amoxicillin	Alone	6.5 ± 0.707	0.344
	A + S	6.5 ± 0.707	
	A + C	7.0 ± 1.414	
	A + M	5.5 ± 0.707	
	A + O	8.5 ± 0.707	
	A + E	10.5 ± 6.364	
	A + G	11.0 ± 1.414	
Streptomycin	Alone	7.5 ± 0.707	0.266
	S + A	6.5 ± 0.707	
	S + C	8.5 ± 0.707	
	S + M	8.5 ± 3.535	
	S + O	9.5 ± 0.707	
	S + E	10.0 ± 1.414	
	S + G	10.5 ± 0.707	

*p* < 0.05 indicate significant difference. C—co-amoxiclav; E—enrofloxacin; A—amoxicillin; S—streptomycin; M—metronidazole; O—oxytetracycline; G—gentamicin.

**Table 5:** Synergy testing of drugs against *E. coli* isolates.

Combinations	MIC AB	MIC A	FIC A	MIC BA	MIC B	FIC B	FICI	Results
amoxi + co-amoxiclav	23.4375	15.625	1.5	5.859375	7.8125	0.75	2.25	Indifferent
amoxi + metro	7.8125	15.625	0.5	62.5	187.5	0.333333	0.833333	Additive
amoxi + enro	5.859375	15.625	0.375	0.976563	1.513672	0.645161	1.020161	Indifferent
amoxi + strepto	5.859375	15.625	0.375	7.8125	18.22917	0.428571	0.803571	Additive
amoxi + genta	4.557292	15.625	0.291667	1.953125	2.929688	0.666667	0.958333	Additive
amoxi + oxy	31.25	15.625	2	31.25	23.4375	1.333333	3.333333	Indifferent
co-amoxiclav + metro	5.859375	7.8125	0.75	125	187.5	0.666667	1.416667	Indifferent
co-amoxiclav + enro	3.90625	7.8125	0.5	0.976563	1.513672	0.645161	1.145161	Indifferent
co-amoxiclav + strepto	2.929688	7.8125	0.375	15.625	18.22917	0.857143	1.232143	Indifferent
co-amoxiclav + genta	3.90625	7.8125	0.5	0.976563	2.929688	0.333333	0.833333	Additive
co-amoxiclav + oxy	7.8125	7.8125	1	31.25	23.4375	1.333333	2.333333	Indifferent
metro + enro	72.91667	187.5	0.388889	0.488281	1.513672	0.322581	0.71147	Additive
metro + strepto	250	187.5	1.333333	15.625	18.22917	0.857143	2.190476	Indifferent
metro + genta	125	187.5	0.666667	1.953125	2.929688	0.666667	1.333333	Indifferent
metro + oxy	250	187.5	1.333333	31.25	23.4375	1.333333	2.666667	Indifferent
enro + strepto	3.90625	1.513672	2.580645	20.50781	18.22917	1.125	3.705645	Indifferent
enro + genta	0.488281	1.513672	0.322581	1.953125	2.929688	0.666667	0.989247	Additive
enro + oxy	4.557292	1.513672	3.010753	31.25	23.4375	1.333333	4.344086	Antagonistic
strepto + genta	15.625	18.22917	0.857143	3.90625	2.929688	1.333333	2.190476	Indifferent
strepto + oxy	20.50781	18.22917	1.125	15.625	23.4375	0.666667	1.791667	Indifferent
genta + oxy	4.557292	2.929688	1.555556	31.25	23.4375	1.333333	2.888889	Indifferent

MIC—minimum inhibitory concentration; FICI—fractional inhibitory concentration index; amoxi—amoxicillin; enro—enrofloxacin; A—amoxicillin; strpto—streptomycin; metro—metronidazole; oxy—oxytetracycline; genta—gentamicin.

## Data Availability

Data is contained within the article or supplementary material.

## Supplementary Materials

The following are available online at https://www.mdpi.com/article/10.3390/antibiotics10080997/s1, Figure S1: Multiple sequence alignment of *E. coli* 23S ribosomal protein (reference and local isolates PK nucleotide sequences), Figure S2: Phylogenetic tree of *E. coli* 23S ribosomal gene (nucleotide sequences), Figure S3: Motifs locations in gene sequences (1 to 5), sequence of individual motif and *p*-value of motifs in sequence 1 to 5, Figure S4: Structural analysis of 23S *E. coli* ribosomal gene from Pakistan, Table S1: Drug combinations and dosage regimens, Table S2: MIC comparison of tested antibiotics against *E. coli*.
