# Supplementary material for: Evidence-Based Tracking of MDR E. coli from Bovine Endometritis and Its Elimination by Effective Novel Therapeutics
Source: Antibiotics (Basel). 2021 Aug 18;10(8):997. doi: 10.3390/antibiotics10080997 (PMC8388920; doi:10.3390/antibiotics10080997)
Supplement: Supplementary file 1 [file antibiotics-10-00997-s001.zip › antibiotics-1319421-supplementary.pdf]

Supplementary Materials

Table S1. Drug combinations and dosage regimens.

Combination Number	Drug A with Dose	Drug B with Dose
i.	Co-amoxiclav (1 mL/10 kg body weight, IM)	Gentamicin (3.3 mg/kg BW, IM)
ii.	Oxytetracycline (17 mg/kg BW)	
iii.	Gentamicin (6.6 mg/15 kg BW, IM)	Enrofloxacin (2.5 mg/kg BW, IM)
iv.	Gentamicin (6.6 mg/15 kg BW, IM)	
v.	Enrofloxacin (20 mL, IU)	Metronidazole (100 mL, IU)
vi.	Amoxicillin (25mL , IU)	Streptomycin (2g, IU)
vii.	Amoxicillin (25mL , IU)	Metronidazole (100 mL, IU)
viii.	Amoxicillin (25mL , IU)	Gentamicin (10mL ,IU)

BW, body weight; IM, intramuscular; IU, Intrauterine.

Table S2. MIC comparison of tested antibiotics against *E. coli.*

Antibiotics	Mean + Std.	95% Confidence Interval for Mean		*p*-value
		Lower Bound	Upper Bound	
**Amoxicillin**	13.54+4.77	8.5322	18.5512	0.0000
**Calamox**	7.70+4.40	3.0819	12.3261	
**Metronidazole**	166.67+75.69	87.2336	246.0998	
**Enrofloxacin**	2.20+1.37	0.7705	3.6403	
**Streptomycin**	11.59+4.11	7.2718	15.9053	
**Gentamicin**	3.02+2.55	0.3447	5.6937	
**Oxytetracycline**	17.07+7.78	8.9102	25.2333	

```
Seq     ATCAACCGAGATTCCCCCAGTAGCGGCGAGCGAACGGGGAGGAGCCCAGAGCCTGAATCA    60
Seq1    ATCAACCGAGATTCCCCCAGTAGCGGCGAGCGAACGGGGAGGAGCCCAGAGCCTGAATCA    60
Seq2    ATCAACCGAGATTCCCCCAGTAGCGGCGAGCGAACGGGGAGGAGCCCAGAGCCTGAATCA    60
Seq3    ATCAACCGAGATTCCCCCAGTAGCGGCGAGCGAACGGGGAGGAGCCCAGAGCCTGAATCA    60
Seq4    ATCAACCGAGATTCCCCCAGTAGCGGCGAGCGAACGGGGAGGAGCCCAGAGCCTGAATCA    60
        ************************************************************

Seq     GTGTGTGTGTTAGTGGAAGCGTCTGGAAAGGCGCGATACAGGGTGACAGCCCCGTACA    120
Seq1    GTGTGTGTGTTAGTGGAAGCGTCTGGAAAGGCGCGATACAGGGTGACAGCCCCGTACA    120
Seq2    GTGTGTGTGTTAGTGGAAGCGTCTGGAAAGGCGCGATACAGGGTGACAGCCCCGTACA    120
Seq3    GTGTGTGTGTTAGTGGAAGCGTCTGGAAAGGCGCGATACAGGGTGACAGCCCCGTACA    120
Seq4    GTGTGTGTGTTAGTGGAAGCGTCTGGAAAGGCGCGATACAGGGTGACAGCCCCGTACA    120
        ************************************************************

Seq     CAAAAAAAATGCACATACTGTGAGCTCGATGAGTAGGGCGGGACACGTGGTATCCTGTCT    180
Seq1    CAAAAAAAATGCACATACTGTGAGCTCGATGAGTAGGGCGGGACACGTGGTATCCTGTCT    180
Seq2    CAAAAAAAATGCACATACTGTGAGCTCGATGAGTAGGGCGGGACACGTGGTATCCTGTCT    180
Seq3    CAAAAAAAATGCACATACTGTGAGCTCGATGAGTAGGGCGGGACACGTGGTATCCTGTCT    180
Seq4    CAAAAAAAATGCACATACTGTGAGCTCGATGAGTAGGGCGGGACACGTGGTATCCTGTCT    180
        ************************************************************

Seq     GAATATGGGGGACCATCCTCCAAGGCTAAATACTCCTGACTGACCGATAGTGA    233
Seq1    GAATATGGGGGACCATCCTCCAAGGCTAAATACTCCTGACTGACCGATAGTGA    233
Seq2    GAATATGGGGGACCATCCTCCAAGGCTAAATACTCCTGACTGACCGATAGTGA    233
Seq3    GAATATGGGGGACCATCCTCCAAGGCTAAATACTCCTGACTGACCGATAGTGA    233
Seq4    GAATATGGGGGACCATCCTCCAAGGCTAAATACTCCTGACTGACCGATAGTGA    233
        *****************************************************
```

Figure S1. Multiple sequence alignment of *E. coli* 23S ribosomal protein (reference and local isolates PK nucleotide sequences).

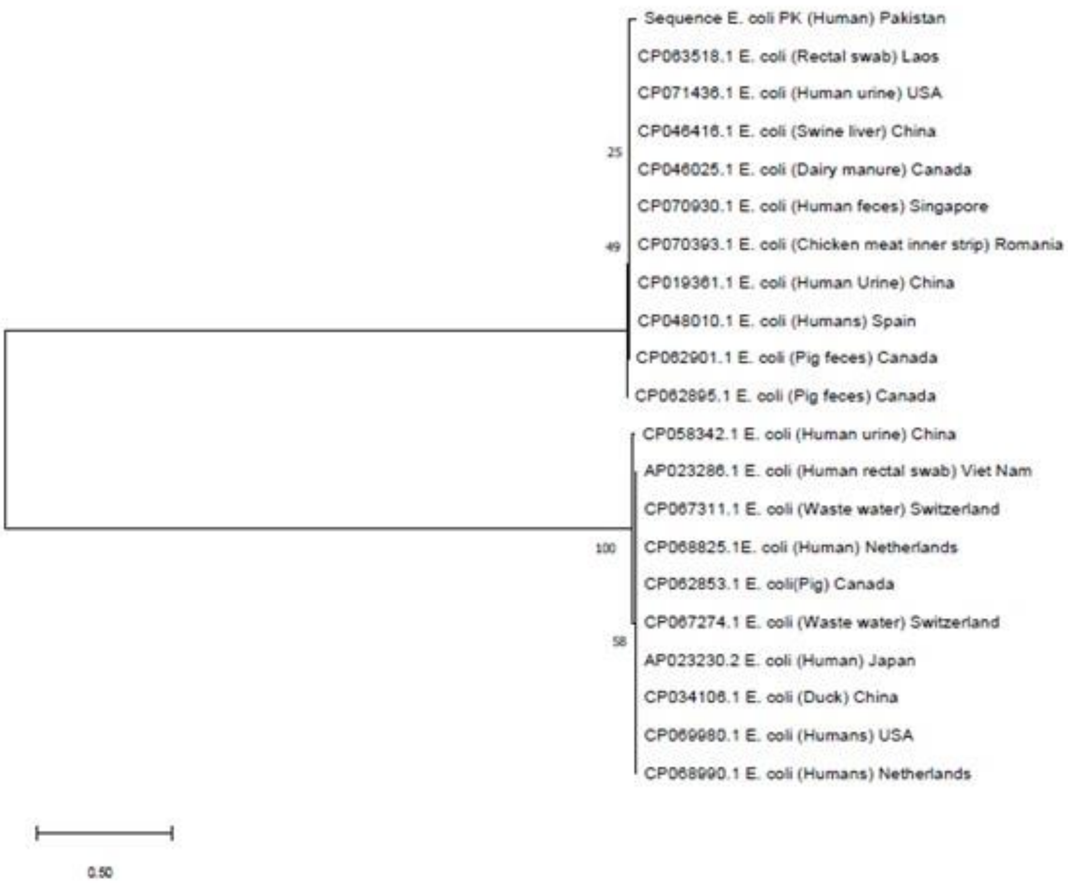

Figure S2. Phylogenetic tree of *E. coli* 23S ribosomal gene (nucleotide sequences). Analysis has been performed among different source samples of *E. coli* 23S ribosomal gene from different countries with our isolated sequenced samples. The branch length (numbers) are representing the nucleotide substitutions per 100 nucleotide sites.

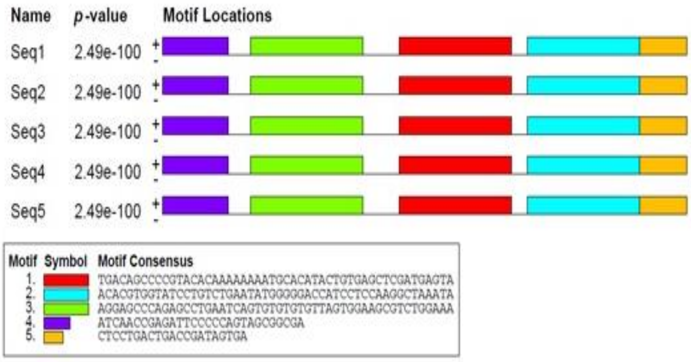

Figure S3. Motifs locations in gene sequences (1 to 5), sequence of individual motif and *p*-value of motifs in sequence 1 to 5.

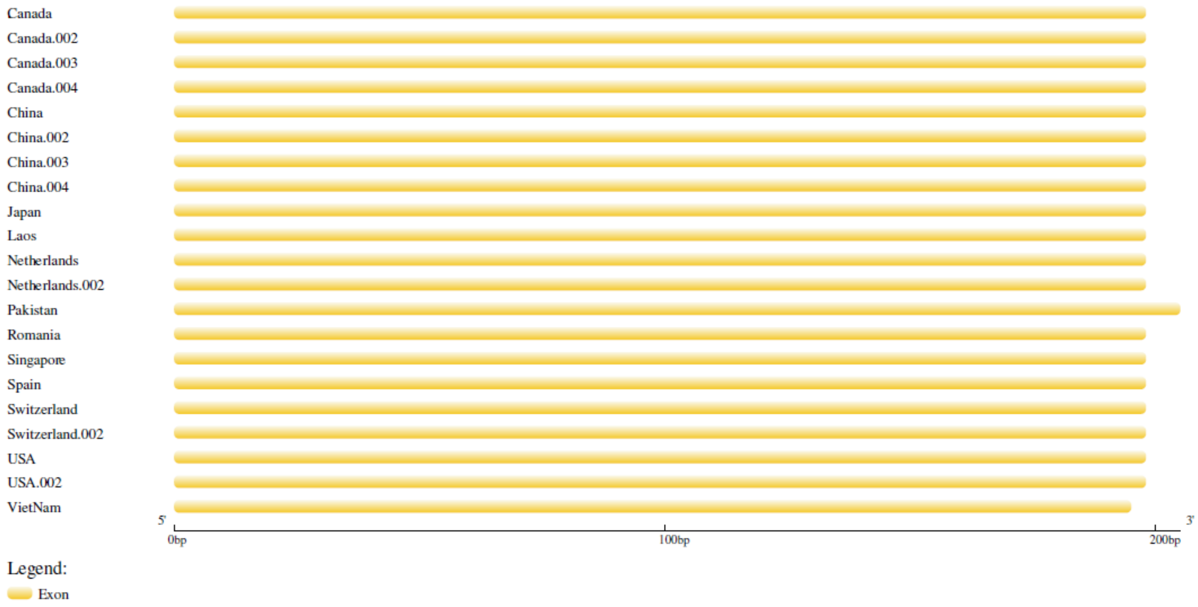

Figure S4. Structural analysis of 23S *E. coli* ribosomal gene from Pakistan.
